# Willingness to provide informal care to older adults in Germany: a discrete choice experiment

**DOI:** 10.1007/s10198-022-01483-5

**Published:** 2022-06-11

**Authors:** Lea de Jong, Torben Schmidt, Jona Theodor Stahmeyer, Sveja Eberhard, Jan Zeidler, Kathrin Damm

**Affiliations:** 1 Center for Health Economics Research Hannover (CHERH), Leibniz University Hannover, Otto-Brenner-Str.7, 30159 Hannover, Germany; 2 Health Services Research Unit, AOK Lower Saxony, Hannover, Germany

**Keywords:** Discrete choice experiment, Elderly care, Older adult care, Long-term care, Preferences, Willingness, C35, I18, J10, J14

## Abstract

As the German population is continually aging and the majority of older adults still wish to ‘age in place’, the need for informal care provided by family and friends will correspondingly continue to increase. In addition, while the need for formal (professional) care services is also likely to increase, the supply already does not meet the demand in Germany today. The aim of our study is the elicitation of people’s willingness to provide informal care by means of a discrete choice experiment. The self-complete postal survey was disseminated to a random sample of the German general population in Lower Saxony. Data cleansing resulted in a final sample size of 280 participants. A conditional logit and a latent class model were estimated. All attributes were judged as highly relevant by the respondents. The results revealed that an increase in the care hours per day had the greatest negative impact overall on the willingness to provide informal care in our sample. The marginal willingness-to-accept for 1 h of informal care was €14.54 when having to provide informal care for 8 h in reference to 2 h per day. This value is considerably higher than the national minimum wage of €9.82. A three-class latent class model revealed preference heterogeneity. While a monetary compensation is often discussed to increase the willingness and availability of informal care in a country, our results show that this statement could not be generalized within our entire sample.

**Supplementary Information:**

The online version contains supplementary material available at 10.1007/s10198-022-01483-5.

## Introduction

Long-term care (LTC) encompasses a variety of services that aim to manage and further delay the functional decline of people with a care dependency by, among others, alleviating pain, assisting with activities of daily living, and ensuring independent living [1]. In Germany, a mandatory LTC insurance was introduced in 1995 to ensure access to LTC services for the entire population. Entitlement to LTC insurance benefits is based on a calculated care dependency grade. For this purpose, a new instrument was introduced in 2017 that uses six modules to determine the need for care of each person on a scale from 0 to 100. The modules and corresponding weights are as follows: 1. Mobility (10%), 2. Cognitive and communicative abilities or 3. Behaviour and psychiatric problems (15%), 4. Self-care (40%), 5. Dealing with requirements due to illness or therapy (20%), and 6. Organisation of everyday life and social contacts (15%). Each module consists of different items for which points are given. In the end, item points are added within each module and incorporated in the final score depending upon the mentioned module weights. The final score is then translated to one of the five care grades. A higher care grade translates to a more severe care dependency and therefore also a higher available budget [2, 3]. Recent statistics show that of the 4.1 million care-dependent Germans in 2019, 80% were cared for at home. Of these, 2.33 million (56%) were cared for exclusively by family members, neighbors, or friends. This type of care is also referred to as informal care and constitutes an important pillar of the LTC system in Germany and many other countries around the world [4].

As the German population is continually aging and the majority of older adults still wish to ‘age in place’, the need for informal care will correspondingly continue to increase. In addition, while the need for home- and community-based services (HCBS) is also likely to increase, the supply already does not meet the demand in Germany today, due to a lack of qualified professionals and care infrastructure [5]. Therefore, recent care reforms have explicitly tried to strengthen home-based care and support informal caregivers, while many still criticize its implementation and reach. Nevertheless, current care systems rely on people’s willingness to provide informal care, as the professional care structures and workforce in place are not able to provide care to the increasing number of care-dependent older adults. When it comes to understanding the reasons for people to take on the role of an informal caregiver, arguments are complex and still not well understood [6]. On the one hand, many studies have highlighted the immense burden informal caregivers’ face in many life settings. First, studies have stressed the toll caregiving can have on a caregiver’s physical and mental health, including back pain, sleep deprivation, and depression [7]. Second, a study by Geyer (2016) has found out that caregivers that provided care for more than 1 h a day needed to reduce their working hours by 5–8 h per week [8]. In addition, re-entering the workforce as full-time employees following the informal caregiving situation is made more difficult [9]. Therefore, informal caregiving can lead to financial hardship [10]. Thirdly, many informal caregivers report that caregiving places increased pressure on their relationships to friends and family. This in turn adds pressure on the informal caregiver, and can lead to self-isolation in some cases and may influence quality of life [11]. On the other hand, studies have also shown that informal caregiving can confer positive psychological effects, which in turn can protect caregivers from experiencing high levels of stress. More specifically, studies report increased levels of resilience, self-confidence, and a sense of meaning [12, 13].

From an economic perspective, the decision to provide informal care is only rational if the utility or value outweighs its costs or burden. Costs or burden can be measured with different methods such as out-of-pocket expenses, time input, or instruments measuring the subjective burden or (health-related) quality of life. Quality of life and well-being instruments can also be used to measure the value of informal care [14]. Several theories try to explain the decision-making process. In an altruistic model, the hypothesis is that the selfless (informal) caregiver draws utility from the well-being of the person in need of care [15]. Cox and Stark (1996) have proposed a different theory, known as the demonstration effect. In this theory, adults with own children are incentivised to provide informal care to their older parents in hopes that their own children internalize the behavior and in turn care for them in the future. Other economic models try to explain informal caregiving using strategic exchanges between the two parties involved. Such exchanges can be in the form of financial incentives or money transfers (e.g., inheritance) between parents and children [16]. To be able to include informal care in economic evaluations, informal care needs to be valued either in terms of costs or carer effects. Monetary valuations of informal care can be done by for instance the opportunity cost method, proxy good method, contingent valuation, conjoint analysis, or discrete choice experiment (DCE), as has been applied in this study [14].

The study on LTC preferences in the field of older adult care has seen an increase over the last few years, especially by means of quantitative stated preference methods, such as DCE, contingent valuation, or best–worst scaling [17]. In the field of LTC, such methods have been used to elicit preferences for different LTC options, the suitability of different LTC settings for hypothetical patient outcomes, as well as the design and structure of specific LTC services such as home-based and community-based services or LTC facilities. Preferences can serve as an important indication to better tailor services to the needs, expectations, and wishes of its consumers. Among choice-based techniques in the field of older adult care, DCE were most often applied and enabled a ranking of the importance of the chosen attributes as well as an assessment on trade-offs respondents were willing to make. Specifically, in the field of informal care, most often contingent valuation methods were applied to explore the value of informal care by estimating the willingness-to-pay (WTP) for a reduction of 1 h in informal caregiving time [18–21]. In one of these studies, willingness-to-accept (WTA) values for having to provide one additional hour of informal care were additionally estimated by informal caregivers in China [21]. In a study by Mentzakis et al. (2011), a DCE was used to value various informal care tasks by informal caregivers in Scotland [22].

To date, one other DCE has been conducted in Germany in the field of LTC, however, focusing on investigating people’s preferences for home- and community-based services [23]. The aim of our study is the elicitation of people’s willingness to provide informal care in Germany by means of a DCE. The use of this methodology not only enables an inquiry into people’s willingness to care but also an assessment of what people value as most important and would be willing to trade-off. As national governments need to establish sustainable and affordable LTC systems, knowledge on people’s willingness to care as well as their trade-offs can add an important puzzle piece for the planning of services as well as support needed to enable more people to take on the role of caregiver.

## Methods

DCEs are increasingly applied in health economics to elicit and quantify people’s preferences. DCEs involve asking respondents to choose between two or more attribute-based alternatives. The underlying assumption of any DCE is that healthcare interventions and products can be decomposed and described by a set of characteristics (attributes) and that people value these differently depending on the levels of each attribute. The discrete choices made by respondents are then analyzed with different regression models and allow the estimation of the relative importance (utility) of each attribute [24, 25].

### Attributes and levels

Results from a systematic literature review of the scientific databases PubMed, Scopus, and Dimdi [26] and 33 semi-structured qualitative interviews [6, 27] were used to identify the most relevant attributes and corresponding levels. While the type and severity of a care dependency as well as the (relationship to) the care-dependent person are important determinants for a person’s willingness to provide care [6], we decided to solely focus on relevant attributes that describe informal caregiving situations in the DCE. Five quantitative attributes with three levels each were identified. A sufficiently wide-level range was classified as is recommended by the literature [28]. The chosen attributes, levels, and the description of each attribute can be found in Table 1. When choosing attributes and levels, compiled choice sets needed to be realistic but also force respondents to trade-off between the levels of each alternative and choose one of the two options. Therefore, 8 h per day in care time was for example chosen as an equivalent to a full-time working day. 0€ per hour of informal care, meaning no financial compensation, was chosen to exemplify the intrinsic willingness to provide care without any monetary compensation. In these scenarios it can be assumed that motivation exists on its own, for instance motivated by love or a sense of obligation for taking care of the relative in need.

**Table 1:** DCE attributes and levels

Attribute	Attribute description	Levels
Expected period of caregiving (duration of care)	The period of time the caregiver would care for and/or look after the person in need of care	6 months 2 years 5 years
Care time (hours per day)	The amount of time (hours per day) the caregiver would provide care and/or supervise the person in need of care at home (e.g., personal care, household tasks, doctor visits etc.)	2 h per day 5 h per day 8 h per day
Formal care services (frequency per week)	The frequency of professional support that is additionally available to the caregiver (e.g., outpatient care services can assist with personal care or counselors can help with any open questions) A visit lasts about 30 min	None 3–4 times a week Daily
Respite (weeks per year)	The number of weeks a year that are available to the caregiver for a variety of respite options. During this time period, professionals care for the individual in need (e.g., during vacation)	None 3 weeks per year 6 weeks per year
Monetary compensation (€ per hour)	A wage replacement benefit (net) at the personal disposal of the caregiver. Paid as a financial compensation per hour for the care provided (in addition to the existing cash benefits by the LTC insurance in Germany)	€0 per hour €6 per hour €12 per hour

The understanding of the attributes and levels as well as the entire questionnaire was piloted in a random sample of the general population (*n* = 30) in a step-wise procedure, meaning that the questionnaire was altered following participant comments and then tested again. The responses led to a series of wording alterations to simplify the questionnaire; however, no attributes or levels had to be changed.

### Experimental design

A two-alternative forced-choice design was created with the software SAS [29]. As the full factorial design would result in 243 (Level Attribute = 3^5^) possible attribute-level combinations, a fractional factorial design with 54 choice sets was created and blocked into six questionnaire versions with nine choice sets each to reduce respondents’ burden. All of these choice sets were checked for plausibility, assigned to the blocks at random and it was ensured that there were no correlated attributes within versions. Generic alternatives (situation A vs. B) were chosen. The fractional factorial design was constructed using the %MktEx macro to make a candidate set of alternatives, followed by the %ChoicEff macro to create an efficient experimental design. The %ChoicEff macro uses a modified Fedorov algorithm, in which all design possibilities are considered and swapped out if the swap improves the D-efficiency [29]. A detailed explanation of all macros can be found in the book by Kuhfeld [29]. A priori attribute coefficients were set to zero in the design. The design with 54 choice sets allowed for the clean estimation of main effects and all two-way interaction effects. In the design construction, the criteria ‘identification’ and ‘efficiency’ were explicitly considered as is recommended by the literature [28]. Identification, meaning that effects can be estimated independently, was determined by the structure of the inverse of the variance–covariance matrix of the parameter estimates. Efficiency, meaning the precision by which effects are estimated, was determined by improving the D-efficiency. The D-efficiency is a standard measure of goodness that can be used to compare the specific experimental designs that are created with the software SAS [29].

### Study population and sample size

The mode of the data collection was a self-complete postal survey. The DCE questionnaire was disseminated to a stratified random sample of 4000 individuals of the German general population via a statutory health insurance (AOK Lower Saxony). The AOK Lower Saxony is the largest health insurance company in its region with a representative structure of insured compared to the entire German population in terms of socio-demographics such as age and sex. Differences were only observed in terms of education and occupation; in particular, the proportion of people with a university degree and higher job complexity was lower among AOK insured compared to the entire Lower Saxony population [30]. A reminder postcard was not sent. The population data from the end of 2017 were used as the data basis to draw a representative sample of the general population by the respective age and gender proportions between 18 and 65 years [31]. We used the equation by Johnson and Orme (2003) to determine the minimum required sample size of 250 respondents [32]. The formula is shown below, where *t* is the number of choice tasks, *a* is the number of alternatives, and *c* is the largest number of levels for any of the attributes or the largest product of levels of any two attributes [32]

$$N >\frac{500c}{(t*a)}$$

### The questionnaire

**Fig. 1:**
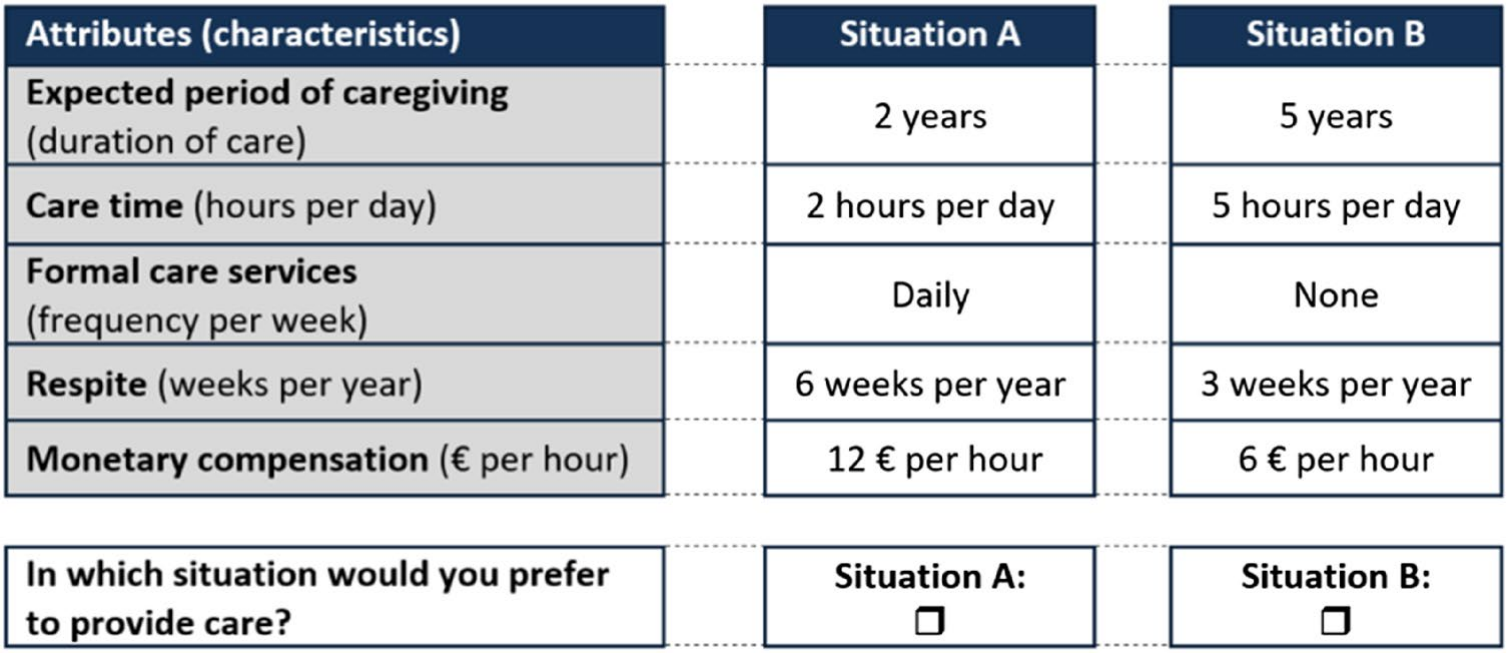
Example of a DCE choice set

The questionnaire was printed in a book format to enable easier readability and was a total of ten pages long. Respondents were given the contact information of the lead author to be able to ask questions. The first page contained a concise participant information including the necessary data protection clarifications. The following two pages enclosed instructions on how to complete the questionnaire, including a table of the attributes and levels as well as an example choice set (see Fig. 1). Respondents were asked to imagine a close relative in need of care. This person was able to be cared for at home and medical or nursing tasks (e.g., wound care) would be cared for professionally. The respondents were then shown an example choice task with two care situations (A vs. B) and it was underlined that no wrong answers were possible, as this was a subjective opinion. The main research question asked to the respondents was: “Under what conditions are you willing to provide care to a close relative? What is important to you personally?”. Subsequently, respondents were asked to choose the preferred care situation in the following nine choice tasks. The questionnaire blocks, meaning the respective DCE choice tasks, were presented to respondents in a random order to ensure that order bias was not systematic across the sample. After the DCE tasks, 19 additional questions were posed regarding age, gender, current health status, living and family situation, income, education, previous caregiving experience, and a number of attitudinal questions regarding the person’s willingness to care (e.g., willingness to reduce working hours). At the end of the questionnaire, a blank space was provided for further comments of the respondents.

### Data analysis and interpretation

Socio-demographic data were analyzed using descriptive methods following data cleansing. Except for mean age, all other variables were depicted as the absolute number of cases and respective percentages in reference to the total sample. The analysis of the collected choice data is theoretically based on Lancaster’s characteristics theory of demand [33] and random utility theory [34]. More specifically, choice data are analyzed on the premise that each individual will choose the alternative (here: care situation) that provides the highest utility to the individual. The utility *U* of individual *q* choosing alternative *i* can be decomposed into a deterministic part *V* and a non-explainable or random component ε and written as [28]

$${U}_{iq}= {V}_{iq}+ {\varepsilon }_{iq}$$

For the multivariate analyses, a conditional logit model (CLM) and a latent class model (LCM) were used. For the CLM, we assumed that error terms are independently distributed with a type 1 extreme value (Gumbel) distribution. Models were estimated without and with two-way interaction effects. The probability of choosing one alternative *i* over the other is given by

$${P}_{iq}= \frac{\mathrm{exp}({V}_{iq})}{{\sum }_{j=1}^{J}\mathrm{exp}({V}_{jq})}$$

All attribute levels were dummy-variable coded, except for the cost attribute in the CLM. Therefore, coefficients were interpreted as deviations from the reference level that was defined beforehand (except for the cost attribute). Positive coefficients > 0 indicate a preference for that attribute level, while negative coefficients < 0 indicate a non-preference for that attribute level. The coefficients were assumed to be statistically significant at a *p* value of ≤ 0.05. Since the DCE included the cost attribute “monetary compensation”, we additionally calculated the marginal WTA (MWTA) for attribute levels in comparison to the reference levels using

$${\mathrm{MWTA}}_{\mathrm{attribute}}=-\left(\frac{{\beta }_{\mathrm{attribute}}}{{\beta }_{\mathrm{cost attribute}}}\right)$$

Further information on the theoretical foundation of DCE can be found elsewhere [22, 28]. In the LCM, we were able to include determining factors (e.g., sex or age) that influenced the choices made in the DCE between care situation A and B (dependent variable). Thus, the LCM allowed an estimation of the importance of DCE attributes for each class as well as the variables that determined class membership to estimate preference heterogeneity. The probability of individual *q* choosing alternative *i* in the depicted situation *t* depending on falling within the class *c* is written as follows:

$${P}_{iqt|c}= \frac{\mathrm{exp} ({x}_{iqt}{\beta }_{c})}{{\sum }_{j=1}^{J}\mathrm{exp}({x}_{jqt}{\beta }_{c})}$$

Models were tested with altering number of classes and different independent variables. Correlations between independent variables were tested using Spearman rank, polychoric, and Cramer’s V correlation measures. As highly correlated independent variables weaken the statistical and explanatory power of our LCM, these were removed. Study population characteristics were included in the segmentation models from the beginning and not analyzed post-classification. For all multivariate analyses, Akaike (AIC) and Bayesian information criteria (BIC), log-likelihood as well as pseudo-R-squared values were used to determine the final model. In particular, when comparing models, the values AIC and BIC should be minimized, while the pseudo-R-squared value should be maximized. All analyses were conducted with R statistics 4.0.4, using the package “survival” for the CLM and the package “lcmm” for the LCM [35].

## Results

### Descriptive statistics

A total of 324 questionnaires were returned (response rate: 8.1%). Of the 324 questionnaires, 44 had missing values with regard to the DCE choice tasks as well as socio-demographic data and were therefore excluded from the analysis. The distribution of age and sex did not significantly differ between the included and the excluded participants, and the missing data were not specific to any one task or socio-demographic question. Socio-demographic data were analyzed descriptively and are shown in Table 2. A considerably higher proportion of women participated in our study (71%). On average, respondents were around 45 years old. The majority of included participants were married or in a permanent relationship (66%) and had children (68%). Around two-thirds of the sample had a high education (at least a completed vocational training or university entrance qualification) and approximately 80% were full- or part-time employed. Almost 60% of the participants had a household income of 1500€ and higher at their disposal. The majority of respondents reported a very good or good health status (65%). Having siblings and the fact whether or not the respondent’s parents were still alive were additionally reported as two factors potentially influencing the reported willingness to provide informal care. 59% of the sample had personal care experiences. This refers to experiences either in organizing informal and/or home-based care services or providing informal care themselves (either alone or with support).

**Table 2:** Characteristics of included participants (*n* = 280)

	*N* = 280
Sex	
Male	81 (29%)
Female	199 (71%)
Mean age (median)	45.2 (49.00)
Marital status	
Single	67 (24%)
Married or in serious relationship	184 (66%)
Widowed	7 (3%)
Divorced or separated	22 (8%)
Having children	
Yes	189 (68%)
No	91 (33%)
Having siblings	
Yes	259 (93%)
No	21 (8%)
Education	
Low	103 (37%)
High	177 (63%)
Current employment status	
Part-time employment	80 (29%)
Full-time employment	133 (48%)
Unemployed	45 (16%)
Retired	22 (8%)
Household income	
Prefer not to say	26 (9%)
Below 500€ up to 1500€	92 (33%)
1500€ up to 3000€	107 (38%)
3000€ to 5000€ and above	55 (20%)
Are your parents still alive?	
Yes, both	134 (48%)
One parent is deceased	84 (30%)
No	62 (22%)
Health status	
Very good	49 (18%)
Good	132 (47%)
Satisfactory	65 (23%)
Less good	27 (10%)
Bad	7 (3%)
Care experience	
Yes	165 (59%)
None	115 (41%)

### Multivariate analyses

#### Conditional logit model

Table 3 shows the main effect coefficients for the CLM and all five attributes were statistically significant to the entire sample. An increase in the expected period of caregiving (duration) as well as the care time per day had a negative impact on respondents’ willingness to care, while the remaining three attributes had a positive impact. The largest negative coefficient was found for having to provide care for 8 h a day in reference to 2 h a day. This indicates that for the entire sample, an increase in the care hours per day reduced willingness to provide informal care. The largest positive coefficient was found for having formal services provide care three to four times a week to the person in need. This indicates that having formal care assistance was very important to the entire sample and increased their willingness to provide care. An increase in the formal care services correspondingly increased the odds of respondents being willing to provide care by the factor of 3.3. The MWTA for 1 h of informal care was €8.77 when having to provide care for 5 h a day and €14.54 when having to provide 8 h of care a day, always in reference to providing care for 2 h a day. For an increase in the expected duration of caregiving, respondents were willing to care for an expected period of 2 years when receiving a minimum of €3.34 of monetary compensation per hour and a minimum of €9.41 for an expected period of 5 years. Negative WTA values indicate that for our entire sample, these attributes or characteristics (formal care services and respite) would result in respondents being willing to forego a monetary compensation or theoretically even additionally pay for these services.

**Table 3:** Conditional logit model (main effects only)

Attributes/levels	Coefficient	OR	95% CI	SE	*P* value	MWTA (€)
Duration (Ref: 6 months)						
2 years	–0.37	0.69	(–0.53; –0.22)	0.08	0.00*	3.34
5 years	–1.06	0.35	(–1.21; –0.90)	0.08	0.00*	9.41
Care time (Ref: 2 h/day)						
5 h/day	–0.99	0.37	(–1.14; –0.83)	0.08	0.00*	8.77
8 h/day	–1.63	0.20	(–1.79; –1.48)	0.08	0.00*	14.54
Formal care services (Ref: none)						
3–4 times/week	1.20	3.31	(1.05; 1.35)	0.08	0.00*	–10.67
Daily	1.14	3.12	(0.98; 1.30)	0.08	0.00*	–10.14
Respite (Ref: none)						
3 weeks/year	0.58	1.78	(0.42; 0.73)	0.08	0.00*	–5.13
6 weeks/year	0.50	1.65	(0.35; 0.66)	0.08	0.00*	–4.48
Monetary compensation (€/h)	0.11	1.12	(0.60; 0.75)	0.01	0.00*	
Log likelihood	–2406.9					
Pseudo-*R*^2^	0.19873					
AIC	4831.8					
BIC	4884.2					
No of observations	5030					
No of coefficients	9					

*OR* odds ratio; *AIC* akaike information criteria; *BIC* bayesian information criteria; *SE* standard error; *MWTA* marginal willingness to accept (€/h)

*p < 0.05

While interaction models are not often applied in the literature due to its complexity, we additionally calculated a CLM with main effects as well as all two-way interaction effects. Results are shown in Table 1 in the supplementary material. Similar to the findings of Nicolet et al. (2018), we found that including all two-way interaction effects slightly improved model fit [36]. In the interaction model, it is important to refrain from interpreting isolated main effects from interaction effects, as such interpretations can be misleading. Additionally, only statistically significant effects can be interpreted. While the main effects for daily formal care services and respite are no longer statistically significant on their own, the interaction effects indicate a very high (positive) impact on peoples’ willingness to provide informal care when care situations included daily formal assistance and respite.

### Latent class model

Models are estimated with different number of classes (2–5) and compared with reference to three goodness-of-fit measures (log-likelihood, AIC, and BIC). A particular emphasis is placed on the BIC when comparing LCMs, as is recommended in the literature [37]. Preference heterogeneity was investigated in reference to seven independent variables by means of the LCM. Based on BIC, a three-class LCM was selected. Class 1 comprised 40% of our sample, class 2 roughly 24%, and class 3 approximately 36%. Table 4 presents an overview of the preference data separated for the three classes.

**Table 4:** Latent class model (attribute preferences)

Attribute/level	Class 1 (*n* = 112, 40%)			Class 2 (*n* = 66, 23.57%)			Class 3 (*n* = 102, 36.43%)		
	Coefficient	SE	*P* value	Coefficient	SE	*P* value	Coefficient	SE	*P* value
Intercept	Not estimated			–1.03	0.18	0.00*	–2.27	0.12	0.00*
Duration (Ref: 6 months)									
2 years	–0.20	0.06	0.00*	–0.59	0.09	0.00*	0.10	0.07	0.12
5 years	–0.68	0.06	0.00*	–1.31	0.10	0.00*	0.14	0.06	0.03*
Care time (Ref: 2 h/day)									
5 h/day	–1.21	0.06	0.00*	0.23	0.10	0.02*	–0.25	0.06	0.00*
8 h/day	–1.83	0.07	0.00*	–0.13	0.09	0.12	–0.37	0.07	0.00*
Formal care services (Ref: none)									
3–4 times/week	0.58	0.06	0.00*	0.68	0.10	0.00*	0.71	0.07	0.00*
Daily	0.20	0.06	0.00*	0.96	0.10	0.00*	0.72	0.07	0.00*
Respite (Ref: none)									
3 weeks/year	0.19	0.06	0.00*	0.30	0.10	0.00*	0.27	0.07	0.00*
6 weeks/year	0.20	0.06	0.00*	0.21	0.09	0.02*	0.41	0.06	0.00*
Monetary compensation (Ref: 0€/h)									
6€/h	0.08	0.06	0.19	0.16	0.09	0.08	0.93	0.07	0.00*
12€/h	0.23	0.06	0.00*	0.10	0.09	0.28	1.66	0.08	0.00*
Log-likelihood	–2617.07								
AIC	5376.14								
BIC	5634.21								
Number of parameters	71								

*p < 0.05

Class 1 (*n* = 112) showed a strong dislike for an increase in the care time per day compared to the remaining two classes. Having to provide care for 8 h compared to 2 h a day had the greatest impact on respondents’ willingness to care (*ß* = –1.82714, *p* < 0.001) in class 1. An increase in the expected period of caregiving (duration) was also valued negatively, while formal care services, respite and 12€ of monetary compensation per hour had a positive impact on the willingness to care of respondents in class 1. In comparison, for class 2 (*n* = 66), an increase in the expected period of caregiving had the greatest (negative) impact on their willingness to provide informal care, in particular 5 years in comparison to 6 months (*ß* = −1.30519, *p* < 0.001). A care time of 5 h in comparison to 2 h a day was valued positively by the respondents of class 2, along with formal care services and respite. Daily formal care services had the greatest positive impact on their willingness to care (*ß* = 0.96246, *p* < 0.001). Monetary compensation had no significant impact on respondents’ decision-making in class 2. Class 3 (*n* = 102) was the only group that valued an expected period of caregiving of 5 years positively (ß = 0.13638, *p* < 0.05). Having to provide care for 8 h a day had a negative impact on the group’s willingness to provide care (*ß* = –0.37385, *p* < 0.001). The most important attribute for class 3 was the monetary compensation. Receiving 12€ per hour of informal care had the greatest positive impact on their willingness to care (*ß* = 1.66179, *p* < 0.001).

As we included several independent variables in the segmentation process of the LCM, class membership effects could additionally be estimated. The differences between class 1 and 2 in reference to the included seven independent variables are shown in Table 5 (referenced against class 3).

**Table 5:** Class membership effects for the latent class model (fixed effects)

	Class 1 (*n* = 112, 40%)			Class 2 (*n* = 66, 23.57%)		
	Coefficient	SE	*P* value	Coefficient	SE	*P* value
Intercept	0.20	0.66	0.77	0.36	0.80	0.65
Sex (Ref: female)	–0.03	0.37	0.95	–1.37	0.55	0.01*
Age group 1 < 35 years (Ref: age group 3 > 50 years)	0.61	0.52	0.24	0.38	0.67	0.58
Age group 2 ≥ 35 and < 50 years (ref: age group 3 > 50 years)	0.59	0.46	0.20	0.87	0.60	0.15
Health status: very good (Ref: satisfactory)	0.47	0.88	0.59	–6.05	33.29	0.86
Health status: good (Ref: satisfactory)	–0.07	0.69	0.92	1.28	0.78	0.10
Health status: less good (Ref: satisfactory)	0.54	0.44	0.22	0.70	0.58	0.23
Health status: bad (Ref: satisfactory)	0.73	0.56	0.20	0.45	0.72	0.54
Having children (Ref: None)	0.04	0.45	0.93	–0.30	0.58	0.61
Household income: prefer not to say (Ref: 1500 up to 3000€)	–0.32	0.58	0.58	–1.01	0.82	0.22
Household income: < 500€ up to < 1500€ (Ref: 1500 up to 3000€)	–0.57	0.41	0.16	–0.53	0.52	0.30
Household income: 3000 to 5000€ and above (Ref: 1500 up to 3000€)	1.23	0.54	0.02^*^	1.23	0.64	0.05
Wishes^a^ for having family provide informal care 1 (Ref: 3)	0.38	0.74	0.61	0.04	0.96	0.97
Wishes^a^ for having family provide informal care 2 (Ref: 3)	0.32	0.75	0.67	1.45	0.80	0.07
Wishes^a^ for having family provide informal care 4 (Ref: 3)	–0.45	0.48	0.35	–0.27	0.56	0.63
Wishes^a^ for having family provide informal care 5 (Ref: 3)	–1.25	0.44	0.00^*^	–1.99	0.66	0.00^*^
Care experience (Ref: none)	–0.32	0.36	0.38	–0.93	0.46	0.04^*^

^*^p < 0.05

^a^Wishes were ranked on a 5-point Likert scale, 1 not important and 5 very important

Respondents in class 1 and 2 did not significantly differ in terms of age and health status in comparison to participants in class 3. Class 2 is comprised of a significantly greater proportion of women (*ß* = –1.37013, *p* < 0.05) and fewer people with care experience compared to class 3 (*ß* = –0.92833, *p* < 0.05). Both classes had a lower proportion of individuals that found it very important (Likert scale: 5) for family members to take care of themselves in case of a care dependency compared to class 3 (class 1: *ß* = –1.24867, *p* < 0.05, class 2: *ß* = –1.99153, *p* < 0.05). Class 2 additionally had a significantly higher proportion of respondents with a high household income in comparison to class 3 (*ß* = 1.23153, *p* < 0.05). The precise socio-demographic structure of all three classes is shown in Table 2 in the supplementary material with the absolute numbers as well as the probabilities per class.

## Discussion

This study investigated the willingness to provide informal care to older adults among 280 participants of the German general population by means of a DCE. With the help of qualitative interviews as well as a systematic literature review, five distinct aspects (attributes) were defined that influence a person’s willingness to provide informal care. All of the included attributes were found to be statistically significant and thus relevant to the respondents when choosing between two hypothetical care situations. Almost all LTC systems around the world rely heavily on the support of informal caregivers and thus indirectly on the continuing willingness of people to provide informal care to their older or sicker relatives in need [4, 38]. Thus, the availability of informal caregivers is predominately determined by people’s willingness to provide care and the support in place to enable informal caregiving [4]. Against the background of changing family structures, growing geographical distances between family members or the increasing employment rates of women, experts expect the rate of informal care to decrease in the future [39]. However, as many Germans still wish to ‘age in place’ and home-based care is considerably less costly for the state and the social security system, informal care remains an important pillar and research topic of interest. As the funding of the German LTC system is based on mandatory contributions, we chose to survey a sample of the general population. This study perspective as well as methodology used is an important distinction to other studies in the field that have predominately investigated the value of informal care by means of the contingent valuation method and surveying informal caregivers [18, 19, 21, 40].

When looking at the results of the CLM, the attribute care time constituted the most important attribute for the entire study population. As expected, needing to provide more hours of informal care per day was valued negatively. For the availability of informal caregivers in a country, a key determinant is the willingness of individuals to provide the number of care hours required for the care-dependent person [4]. Even though the needed care time per day or the expected duration of caregiving is difficult to plan ahead [6], it is important to know what people can imagine in terms of providing informal care. Studies show that with increasing care dependency, the necessary care time per day is often higher than our maximum level of 8 h care time per day [41, 42]. Nevertheless, the chosen level of 8 h was specifically intended to represent an equivalent to a full working day in Germany to additionally survey a willingness to reduce working hours if necessary. Other studies have also found that an increase in care hours per day often results in the reduction or temporary pause of working hours [8, 10].

One major challenge that is often described is the necessary reconciliation of informal care with other personal responsibilities, such as needing to work to ensure financial stability or having younger children at home to take care of. The heavy burden informal caregivers shoulder as a result of conflicting responsibilities often in turn lead to high physical and mental strain [7, 10]. One economic incentive for informal care provision that is discussed in politics is a monetary compensation paid to informal caregivers to increase peoples’ willingness to care [43]. In Germany, the idea of such a monetary compensation would be paid in addition to the existing insurance benefits available to the care-dependent person, similar to other legal entitlements such as parental leave. Such a monetary compensation might ensure financial stability for the informal caregiver for a period of time by enabling a reduction of working hours [43]. As we included such a financial compensation as one attribute in our DCE, we were able to calculate WTA values for the different attribute levels. The highest WTA value of €14.54 per hour was found when being willing to provide 8 h of care in reference to 2 h of care per day, followed by €9.41 per hour when having to provide care for an estimated period of 5 years instead of six months. The current minimum wage in Germany is €9.82, which is considerably lower than the accepted value of €14.54 per hour of informal caregiving [44]. A similar approach was taken in the DCE by Mentzakis et al. (2011), however, to estimate monetary values for specific informal care tasks such as personal care or household tasks [22]. While several studies have found significant differences between WTA and WTP values [21, 45], a Dutch study by van den Berg et al. (2005) found only minor differences between WTP and WTA when it comes to informal care valuations [46].

Preference heterogeneity was additionally investigated in this study with a three-class LCM. Especially when it comes to the above-mentioned monetary compensation, a higher financial compensation had in fact the highest positive impact on the willingness to provide care of respondents in class 3 (*n* = 102). This could in part be explained by class 3 having a significantly lower household income at their disposal in reference to class 1. Class 1 (*n* = 112) placed the greatest negative weight by far on increasing care hours per day. For class 2 (*n* = 66), instead of care hours, an increase in the expected duration of caregiving had the greatest negative value and the greatest positive impact was found for daily formal care services. Monetary compensation had no significant impact on respondent’s willingness to provide care in class 2. Class 2 had a significantly higher proportion of women and respondents without care experiences compared to class 3. Moreover, wishes in terms of people’s willingness to receive informal care in the future had a significant impact in both classes 1 and 2 in reference to class 3. Both classes had a significantly lower proportion of study participants that found it very important for their relatives to take care of them in the event of a care dependency. Thus, respondents of class 3 seemed to be very willing to provide informal care and in turn would wish for the same willingness by their relatives. While not statistically significant in our study, others have found determining factors for peoples’ willingness to make use of informal care to include having children and living together with a partner [47].

To the best of our knowledge, our study is the first to investigate the willingness to provide informal care of the German general population by means of a DCE. While, in total, studies on LTC preferences in the field of older adult care have seen an increase over the past years, a direct comparison of our results to other studies in the field of informal care is challenging. However, as we included the attribute formal care services, one particular study of interest is the DCE conducted by Lehnert et al. (2018) in Germany. In this DCE, the authors also surveyed a sample of the general population to investigate preferences for home- and community-based formal care services. Two hypothetical care packages were distinguished in reference to five attributes: care time per day, service level, quality of care, number of caregivers per month, and a co-payment per month [23]. The results of the CLM can provide some indications towards the possible preferences or design of the attribute formal care services that was integrated in our DCE. Results of the study by Lehnert et al. (2018) show that very high quality of care and smaller groups of formal caregivers (less rotation) were preferred. The calculated WTP for one extra hour of formal care was €8.98 for the surveyed sample [23].

### Limitations

This study has several limitations that need to be addressed. The sample was only recruited in one federal state of Germany (Lower Saxony), which means that transferability of study findings is limited. In addition, the response rate of 8.1% is considerably lower compared to other studies in the field of informal care (20%, [22]) or home-based care (23.4%, [23]). Unfortunately, a relatively high proportion of questionnaires also had to be excluded due to missing values (44 out of 324). This might be due to the complexity of the chosen method DCE combined with the research topic and postal survey. Due to the limited sample size, it was not appropriate to derive concrete policy suggestions or recommendations. For this reason, future studies should attempt to include a considerably larger and optimally German-wide sample to increase representativeness. Additionally, no reminder was sent in our study, as we believed that this topic of interest either sparked interest in participants or not. As previous qualitative work has shown that willingness to provide informal care is difficult for some to actively deal with until such a situation arises in the family, we believed that a reminder would not significantly increase participation [6]. Moreover, a considerably higher proportion of women participated in our study, which might also be explained by the research topic. We had similar challenges in our qualitative work in the field [6]. A sample selection bias is therefore possible and means interpretation of study results need to be done cautiously.

In the design of the DCE, we were unfortunately not able to integrate changes in the type and severity of the care dependency such as cognitive compared to physical impairments. As we expect this to have an impact on people’s willingness to provide care, this should be integrated in future studies. It needs to be noted that willingness to provide care is additionally influenced by many other factors, such the relationship to the person in need of care, cultural and normative beliefs, as well as surrounding circumstances such as the geographical distance between family members or the available housing space [6]. The interpretation of these influencing factors were consciously left open to each study participant in the DCE as only the five attributes and the context of the care situation were provided. Moreover, the availability, quality, and affordability of near-by formal alternatives such as nursing homes might also impact willingness to provide informal care. This, however, is regionally very different in Germany and difficult to integrate in a DCE without substantially increasing the complexity of the choice sets. The use of a forced-choice design forced respondents to always choose between the two alternatives, even if in reality people might opt out and choose not to provide informal care.

Since our DCE data were only collected at one point in time, no temporal changes in people’s willingness to care could be measured. As qualitative studies have shown that willingness to care is usually influenced by a number of complex contextual factors and can change over time with, for instance, altering personal responsibilities or changes in people’s health status. Future studies should further investigate changes in people’s willingness compared to the actual provision of informal care over time. Nevertheless, as some studies suggest that informal care will likely continue to decrease in the future, while the need for this type of care remains high, it remains important to investigate people’s perceptions and general willingness to provide care. More specifically, it is vital to investigate which factors have a considerable impact on people’s willingness to provide care, such as the included monetary compensation. Unfortunately, several independent variables had too little variation in our sample, which increased correlations between variables and made it impossible to estimate an effect of these variables on the class segmentation of the LCM. A bigger sample might enable the inclusion of further independent variables, such as the employment status in future studies.

## Conclusion

The present study is the first that investigated people’s willingness to provide informal care by means of a DCE. Willingness to provide care was decomposed into five distinct aspects (attributes). With the help of regression models, the relative importance and trade-offs between attributes could be inferred. Under the premise that informal care remains a vital pillar of the German LTC system, results can provide insights into structural aspects that need to be improved to ensure that people are willing to provide informal care without too much mental and physical strain, as this in turn often leads to higher health costs and work absenteeism. The results of our LCM showed that compared to preferences of our entire sample, preferences could be segmented into three distinct groups that placed a different focus on attributes. Care time per day and expected duration of caregiving were valued negatively, however, in the three groups to a significantly different extend. Class 1 placed by far the greatest negative weight on an increase in the care time by day. Class 2 had a lower proportion of people with caregiving experiences and placed the highest value on reducing the expected time period of caregiving as well as having daily formal care services for support. While a monetary compensation is discussed to increase the willingness and availability of informal care in a country, our results show that this statement could not be generalized to our entire sample. More specifically, a monetary compensation might therefore only reach and motivate a sample of the population (here class 3), in particular as our results show people with a lower household income at their disposal.

## Supplementary Information

Below is the link to the electronic supplementary material.

Supplementary file1 (DOCX 20 KB)

## References

[1] OECD/European Commission: OECD health policy studies: a good life in old age? Monitoring and improving quality in long-term care. OECD Publishing (2013). 10.1787/2074319x

[2] Arntz M, Sacchetto R, Spermann A, Steffes S, Widmaier S (2007). The German social long-term care insurance-structure and reform options. SSRN J.

[3] Bundesministerium der Justiz und für Verbraucherschutz. Sozialgesetzbuch: Soziale Pflegeversicherung: § 15 Ermittlung des Grades der Pflegebedürftigkeit, Begutachtungsinstrument. https://www.sozialgesetzbuch-sgb.de/sgbxi/15.html. Accessed March 2

[4] Zigante, V.: Informal care in Europe: exploring formalisation, availability and quality. European Commission, London School of Economics and Political Science (2018). https://data.europa.eu/doi/10.2767/78836. Accessed 25 Feb 2022

[5] Völz, S., Schnecke, J.H.: Beruf und Pflege besser vereinbaren: Individuelle und betriebliche Perspektiven als regionaler Gestaltungsansatz. Forschung Aktuell 03/2021, 1–23 (2021)

[6] de Jong L, Stahmeyer JT, Eberhard S, Zeidler J, Damm K (2021). Willingness and preparedness to provide care: interviews with individuals of different ages and with different caregiving experiences. BMC Geriatr.

[7] Hajek A, Brettschneider C, Ernst A, Posselt T, Wiese B, Prokein J, Weyerer S, Werle J, Fuchs A, Pentzek M (2016). Longitudinal predictors of informal and formal caregiving time in community-dwelling dementia patients. Soc Psychiatry Psychiatr Epidemiol.

[8] Geyer, J.: Informell Pflegende in der deutschen Erwerbsbevölkerung: Soziodemografie, Pflegesituation und Erwerbsverhalten. Zentrum für Qualität in der Pflege (eds.) ZQP-Themenreport. Vereinbarkeit von Beruf und Pflege, pp. 24–43. ZQP, Berlin (2016)

[9] Keck W (2016). Was kommt nach der Pflege? Die Pflege eines Angehörigen senkt Beschäftigungschancen von Pflegepersonen nachhaltig. Sozialer Fortschr.

[10] Geyer J, Schulz E (2014). Who cares? Die Bedeutung der informellen Pflege durch Erwerbstätige in Deutschland. Diw Wochenber.

[11] Rothgang H, Müller R, Runte R, Unger R (2018). Pflegereport 2018 Schriftenreihe zur Gesundheitsanalyse.

[12] Stansfeld J, Stoner CR, Wenborn J, Vernooij-Dassen M (2017). Positive psychology outcome measures for family caregivers of people living with dementia: a systematic review. Int Psychogeriatr.

[13] Pendergrass A, Mittelman M, Graessel E, Özbe D, Karg N (2019). Predictors of the personal benefits and positive aspects of informal caregiving. Aging Ment Health.

[14] Hoefman RJ, van Exel J, Brouwer W (2013). How to include informal care in economic evaluations. Pharmacoeconomics.

[15] Schneider U (2006). Informelle Pflege aus ökonomischer Sicht. Zeitschr Sozialreform.

[16] Jiménez-Martín S, Vilaplana Prieto C (2015). Informal care motivations and intergenerational transfers in European countries. Health Econ.

[17] Lehnert T, Heuchert MA, Hussain K, Koenig H-H (2019). Stated preferences for long-term care: a literature review. Ageing Soc.

[18] Gervès C, Bellanger MM, Ankri J (2013). Economic analysis of the intangible impacts of informal care for people with Alzheimer’s disease and other mental disorders. Value Health.

[19] Gustavsson A, Jönsson L, McShane R, Boada M, Wimo A, Zbrozek AS (2010). Willingness-to-pay for reductions in care need: estimating the value of informal care in Alzheimer’s disease. Int J Geriatr Psychiatry.

[20] König M, Wettstein A (2002). Caring for relatives with dementia: willingness-to-pay for a reduction in caregiver’s burden. Expert Rev Pharm Outcomes Res.

[21] Liu W, Lyu T, Zhang X, Yuan S, Zhang H (2020). Willingness-to-pay and willingness-to-accept of informal caregivers of dependent elderly people in Shanghai, China. BMC Health Serv Res.

[22] Mentzakis E, Ryan M, McNamee P (2011). Using discrete choice experiments to value informal care tasks: exploring preference heterogeneity. Health Econ.

[23] Lehnert T, Günther OH, Hajek A, Riedel-Heller SG, König HH (2018). Preferences for home-and community-based long-term care services in Germany: a discrete choice experiment. Eur J Health Econ.

[24] Johnson FR, Lancsar E, Marshall D, Kilambi V, Mühlbacher A, Regier DA, Bresnahan BW, Kanninen B, Bridges JFP (2013). Constructing experimental designs for discrete-choice experiments: report of the ISPOR conjoint analysis experimental design good research practices task force. Value Health.

[25] Mühlbacher AC, Bethge S, Tockhorn A (2013). Präferenzmessung im gesundheitswesen: grundlagen von discrete-choice-experimenten. Gesundheitsökon Qualitätsmanage.

[26] Plöthner M, Schmidt K, de Jong L, Zeidler J, Damm K (2019). Needs and preferences of informal caregivers regarding outpatient care for the elderly: a systematic literature review. BMC Geriatr.

[27] de Jong L, Stahmeyer JT, Eberhard S, Zeidler J, Damm K (2020). „Aber vielfach scheitert man dann an Besonderheiten “–Pflegeberater über Gesetzesänderungen und die Herausforderungen ihrer Arbeit: Eine qualitative Untersuchung. Z Evid Fortbild Qual Gesundhwes.

[28] Lancsar E, Louviere J (2008). Conducting discrete choice experiments to inform healthcare decision making. Pharmacoeconomics.

[29] SAS Institute Inc. (ed.): Marketing research methods in SAS: experimental design, choice, conjoint, and graphical techniques. SAS Document TS-694.http://support.sas.com/techsup/technote/ts694.pdf. Accessed on March 2. Citeseer (2007)

[30] Epping J, Geyer S, Eberhard S, Tetzlaff J (2021). Völlig unterschiedlich oder doch recht ähnlich? Die soziodemografische Struktur der AOK Niedersachsen im Vergleich zur niedersächsischen und bundesweiten Allgemein-und Erwerbsbevölkerung. Das Gesundheitswesen.

[31] Destatis—Statistisches Bundesamt: Bevölkerungsstand—Bevölkerung nach Altersgruppen. https://www.destatis.de/DE/Themen/Gesellschaft-Umwelt/Bevoelkerung/Bevoelkerungsstand/Tabellen/bevoelkerung-altersgruppen-deutschland.html. Accessed 8 October 2021

[32] Johnson, R., Orme, B.: Getting the most from CBC. Sawtooth Software Research Paper Series, Sequim, Sawtooth Software (2003)

[33] Lancaster KJ (1966). A new approach to consumer theory. J Polit Econ.

[34] Manski CF (1977). The structure of random utility models. Theor Decis.

[35] The R Foundation for Statistical Computing: The R Foundation. https://www.r-project.org/foundation/. Accessed 19 October 2021

[36] Nicolet A, Groothuis-Oudshoorn CGM, Krabbe PFM (2018). Does inclusion of interactions result in higher precision of estimated health state values?. Value health.

[37] Nylund KL, Asparouhov T, Muthén BO (2007). Deciding on the number of classes in latent class analysis and growth mixture modeling: a Monte Carlo simulation study. Struct Equ Model.

[38] Spasova S, Baeten R, Vanhercke B (2018). Challenges in long-term care in Europe. Eurohealth.

[39] Hajek A, Lehnert T, Wegener A, Riedel-Heller SG, König H-H (2018). Potential for informal care of the elderly in Germany results of a representative population-based survey (potential for informal care of the elderly in Germany: results of a representative population-based survey). Zeitschr Gerontol Geriatr.

[40] Fu YY, Chui EW, Law CK, Zhao X, Lou VWQ (2019). An exploration of older Hong Kong residents’ willingness to make copayments toward vouchers for community care. J Aging Soc Policy.

[41] Wetzstein M, Rommel A, Lange C (2015). Pflegende Angehörige-Deutschlands größter Pflegedienst. GBE kompakt.

[42] Hielscher, V., Kirchen-Peters, S., Nock, L., Ischebeck, M.: Pflege in den eigenen vier Wänden: Zeitaufwand und Kosten. Pflegebedürftige und ihre Angehörigen geben Auskunft. (eds.) Hans-Böckler-Stiftung, No. 263. Hans-Böckler-Stiftung, Düsseldorf (2017)

[43] Rothgang H (2019). Stellungnahme zum Antrag der Fraktion Bündnis 90/Die Grünen „Pflege gerecht und stabil finanzieren–Die Pflege-Bürgerversicherung vollenden“. BT-Drucksache.

[44] Destatis—Statistisches Bundesamt: Verdienste—Mindestlohn. https://www.destatis.de/DE/Themen/Arbeit/Verdienste/Mindestloehne/_inhalt.html (2022). Accessed 11 January 2022

[45] Horowitz JK, McConnell KE (2002). A review of WTA/WTP studies. J Environ Econ Manage.

[46] van den Berg B, Bleichrodt H, Eeckhoudt L (2005). The economic value of informal care: a study of informal caregivers' and patients' willingness to pay and willingness to accept for informal care. Health Econ.

[47] Spangenberg L, Glaesmer H, Brähler E, Strauß B (2012). Use of family resources in future need of care preferences and expected willingness of providing care among relatives: a population-based study. Bundesgesundheitsbl.

