# Supplementary material for: Willingness to provide informal care to older adults in Germany: a discrete choice experiment
Source: Eur J Health Econ. 2022 Jun 11;24(3):425–36. doi: 10.1007/s10198-022-01483-5 (PMC10060358; doi:10.1007/s10198-022-01483-5)
Supplement: Supplementary file 1 — Supplementary file1 (DOCX 20 KB) [file 10198_2022_1483_MOESM1_ESM.docx]

**Supplementary material**

Table 1: Conditional logit model (*main effects + all two-way interaction effects*)

| **Attribute/Level** | **Coefficient** | **OR** | **95% CI** | **SE** | ***p*-value** |
| --- | --- | --- | --- | --- | --- |
| Duration (*Ref: 6 months*) |  |  |  |  |  |
| 2 years | 1.44 | 4.23 | (0.75; 2.13) | 0.35 | 0.00* |
| 5 years | –0.46 | 0.63 | (–0.92; –0.01) | 0.23 | 0.04* |
| Care time (*Ref: 2 hours/day*) |  |  |  |  |  |
| 5 hours/day | –1.12 | 0.33 | (–1.64; –0.59) | 0.27 | 0.00* |
| 8 hours/day | –2.02 | 0.13 | (–2.63; –1.41) | 0.31 | 0.00* |
| Formal care services (*Ref: None*) |  |  |  |  |  |
| 3–4 times/week | 1.43 | 4.19 | (0.93; 1.94) | 0.26 | 0.00* |
| Daily | –0.48 | 0.62 | (–1.06; 0.11) | 0.30 | 0.11 |
| Respite (*Ref: None*) |  |  |  |  |  |
| 3 weeks/year | 0.06 | 1.06 | (–0.47; 0.59) | 0.27 | 0.83 |
| 6 weeks/year | 0.05 | 1.05 | (–0.55; 0.65) | 0.31 | 0.88 |
| Monetary compensation (€/hour) | 1.05 | 2.87 | (0.77; 1.33) | 0.14 | 0.00* |
| *Interaction effects* |  |  |  |  |  |
| 2 years x 5 hours/day | –0.39 | 0.68 | (–0.91; 0.13) | 0.27 | 0.14 |
| 2 years x 8 hours/day | –1.41 | 0.24 | (–2.03; –0.78) | 0.32 | 0.00* |
| 2 years x 3–4 times/week | –1.00 | 0.37 | (–1.54; –0.45) | 0.28 | 0.00* |
| 2 years x Daily | –0.45 | 0.63 | (–1.05; 0.14) | 0.30 | 0.14 |
| 2 years x 3 weeks/year | –1.09 | 0.34 | (–1.79; –0.38) | 0.36 | 0.00* |
| 2 years x 6 weeks/year | –0.87 | 0.42 | (–1.47; –0.28) | 0.30 | 0.00* |
| 2 years x Monetary compensation | –0.49 | 0.61 | (–0.75; –0.22) | 0.13 | 0.00* |
| 5 years x 5 hours/day | –0.53 | 0.59 | (–1.02; –0.04) | 0.25 | 0.04* |
| 5 years x 8 hours/day | –1.01 | 0.36 | (–1.55; –0.48) | 0.27 | 0.00* |
| 5 years x 3–4 times/week | –0.71 | 0.49 | (–1.27; –0.15) | 0.29 | 0.01* |
| 5 years x Daily | 0.55 | 1.73 | (0.06; 1.04) | 0.25 | 0.03* |
| 5 years x 3 weeks/year | –0.20 | 0.82 | (–0.74; 0.33) | 0.27 | 0.45 |
| 5 years x 6 weeks/year | 0.20 | 1.23 | (–0.36; 0.77) | 0.29 | 0.48 |
| 5 years x Monetary compensation | –0.39 | 0.67 | (–0.62; –0.17) | 0.12 | 0.00* |
| 5 hours/day x 3–4 times/week | 0.62 | 1.87 | (0.18; 1.07) | 0.23 | 0.01* |
| 5 hours/day x Daily | 0.75 | 2.11 | (0.14; 1.36) | 0.31 | 0.02* |
| 5 hours/day x 3 weeks/year | 0.28 | 1.32 | (–0.25; 0.80) | 0.27 | 0.30 |
| 5 hours/day x 6 weeks/year | 0.16 | 1.18 | (–0.36; 0.69) | 0.27 | 0.54 |
| 5 hours/day x Monetary compensation | –0.23 | 0.79 | (–0.49; 0.02) | 0.13 | 0.08 |
| 8 hours/day x 3–4 times/week | 0.78 | 2.19 | (0.14; 1.43) | 0.33 | 0.02* |
| 8 hours/day x Daily | –0.74 | 0.48 | (–1.46; –0.02) | 0.37 | 0.04* |
| 8 hours/day x 3 weeks/year | 0.99 | 2.70 | (0.42; 1.57) | 0.29 | 0.00* |
| 8 hours/day x 6 weeks/year | 1.19 | 3.30 | (0.60; 1.79) | 0.30 | 0.00* |
| 8 hours/day x Monetary compensation | –0.26 | 0.77 | (–0.56; 0.05) | 0.16 | 0.10 |
| 3–4 times/week x 3 weeks/year | 0.21 | 1.24 | (–0.36; 0.78) | 0.29 | 0.46 |
| 3–4 times/week x 6 weeks/year | –0.32 | 0.73 | (–0.82; 0.18) | 0.25 | 0.20 |
| 3–4 times/week x Monetary compensation | –0.03 | 0.97 | (–0.29; 0.22) | 0.13 | 0.80 |
| Daily x 3 weeks/year | 2.41 | 11.18 | (1.82; 3.01) | 0.30 | 0.00* |
| Daily x 6 weeks/year | 1.55 | 4.70 | (0.99; 2.11) | 0.29 | 0.00* |
| Daily x Monetary compensation | 0.21 | 1.23 | (–0.06; 0.48) | 0.14 | 0.13 |
| 3 weeks/year x Monetary compensation | –0.07 | 0.93 | (–0.31; 0.16) | 0.12 | 0.55 |
| 6 weeks/year x Monetary compensation | –0.16 | 0.85 | (–0.42; 0.11) | 0.13 | 0.24 |
| Log likelihood | –2296.7 |  |  |  |  |
| Pseudo R^2^ | 0.22467 |  |  |  |  |
| AIC | 4675.4 |  |  |  |  |
| BIC | 4914.4 |  |  |  |  |
| No. of observations | 5030 |  |  |  |  |
| No. of coefficients | 41 |  |  |  |  |

**significant at p<0.05, Monetary compensation is standardized*

Table 2: Socio-demographic structure of the classes within the LCM

|  | **Class 1 (n = 122)** | **Class 2 (n = 66)** | **Class 3 (n = 102)** |
| --- | --- | --- | --- |
| Sex |  |  |  |
| Male | 38 (33.93) | 9 (13.64) | 34 (33.33) |
| Female | 74 (66.07) | 57 (86.36) | 68 (66.67) |
| Age group |  |  |  |
| <35 years | 37 (33.06) | 16 (24.24) | 23 (22.55) |
| ≥35 & <50 years | 29 (25.89) | 22 (33.33) | 18 (17.65) |
| >50 years | 46 (41.07) | 28 (42.42) | 61 (59.80) |
| Health status |  |  |  |
| Very good | 26 (23.21) | 10 (15.15) | 13 (12.75) |
| Good | 55 (49.11) | 34 (51.51) | 43 (42.16) |
| Satisfactory | 23 (20.54) | 12 (18.18) | 30 (29.41) |
| Less good | 5 (4.46) | 10 (15.15) | 12 (11.76) |
| Bad | 3 (2.68) | 0 (0.00) | 4 (3.92) |
| Having children |  |  |  |
| Yes | 73 65.18) | 44 (66.67) | 72 (70.59) |
| No | 39 (34.82) | 22 (33.33) | 30 (29.41) |
| Household income |  |  |  |
| Prefer not to say | 10 (8.93) | 4 (6.06) | 12 (11.76) |
| <500€ – 1500€ | 27 (24.11) | 20 (30.30) | 45 (44.12) |
| 1500€ – 3000€ | 45 (40.18) | 24 (36.36) | 38 (37.25) |
| 3000€ – 5000€ and more | 30 (26.79) | 18 (27.27) | 7 (6.86) |
| Wishes |  |  |  |
| 1 (not important) | 9 (8.04) | 3 (4.55) | 5 (4.90) |
| 2 | 8 (7.14) | 12 (18.18) | 4 (3.92) |
| 3 | 37 (33.04) | 22 (33.33) | 19 (18.63) |
| 4 | 29 (25.89) | 19 (28.79) | 20 (19.61) |
| 5 (very important) | 29 (25.89) | 10 (15.15) | 54 (52.94) |
| Care experiences |  |  |  |
| Yes | 71 (63.39) | 31 (46.97) | 63 (61.76) |
| No | 41 (36.61) | 35 (53.03) | 39 (38.24) |

*Note: The absolute and relative numbers per class are shown.*
